# Right ventricular size and function under riociguat in pulmonary arterial hypertension and chronic thromboembolic pulmonary hypertension (the RIVER study)

**DOI:** 10.1186/s12931-018-0957-y

**Published:** 2018-12-19

**Authors:** Alberto M. Marra, Michael Halank, Nicola Benjamin, Eduardo Bossone, Antonio Cittadini, Christina A. Eichstaedt, Benjamin Egenlauf, Satenik Harutyunova, Christine Fischer, Henning Gall, Hossein Ardeschir Ghofrani, Marius M. Hoeper, Tobias J. Lange, Karen M. Olsson, Hans Klose, Ekkehard Grünig

**Affiliations:** 1 IRCCS SDN, Research Institute, Naples, Italy; 2 Centre for Pulmonary Hypertension, Thoraxklinik at Heidelberg University Hospital, Röntgenstraße 1, 69126 Heidelberg, Germany; 3 Translational Lung Research Center Heidelberg (TLRC), Member of the German Center for Lung Research (DZL), Heidelberg, Germany; 4 Department of Internal Medicine I, Pneumology, University Hospital Dresden, Dresden, Germany; 5 Cardiology Division, A. Cardarelli Hospital, Naples, Italy; 6 Department of Translational Medical Sciences, “Federico” University, Naples, Italy; 7 Institute of Human Genetics, University Hospital Heidelberg, Heidelberg, Germany; 8 Department of Internal Medicine, Justus-Liebig-University Giessen, Universities of Giessen and Marburg Lung Center (UGMLC), Member of the German Center for Lung Research (DZL), Giessen, Germany; 9 Department of Respiratory Medicine, Member of the German Center for Lung Research (DZL), Hannover Medical School, Hannover, Germany; 10 Department of Internal Medicine II, Division of Pneumology, University Medical Center Regensburg, Regensburg, Germany; 11 Department of Pneumology, University Hospital Hamburg-Eppendorf, Hamburg, Germany

**Keywords:** Pulmonary hypertension, Pulmonary arterial hypertension, Chronic thromboembolic pulmonary hypertension, Riociguat, Soluble guanylate cyclase stimulator, Right atrial area, Right heart size, Right ventricular function, Echocardiography

## Abstract

**Background:**

Riociguat is a soluble guanylate cyclase stimulator approved for pulmonary arterial hypertension (PAH) and chronic thromboembolic pulmonary hypertension (CTPEH). The objective of this study was to evaluate right heart size and function assessed by echocardiography during long term treatment with riociguat.

**Methods:**

Patients who started riociguat treatment (1.0–2.5 mg tid) within the trials phase II, PATENT, PATENTplus, EAS, CHEST and continued treatment for 3–12 months were included in this study. Echocardiography was analysed off-line at baseline, after 3, 6 and 12 months by investigators who were blinded to clinical data. Last and baseline observation carried forward method (LOCF, BOCF) were performed as sensitivity analysis.

**Results:**

Seventy-one patients (45% PAH, 55% CTEPH; 53.5% female; 60 ± 13 years, mean pulmonary arterial pressure 46 ± 10 mmHg, mean PVR 700 ± 282dynes·sec·cm-5) were included. After 6 months, RA and RV area, RV thickness tricuspid regurgitation velocity showed a significant reduction. After 12 months, patients receiving riociguat therapy showed a significant reduction in right atrial (− 2.6 ± 4.4 cm2, 95% CI -3.84, − 1.33; *p* < 0.001, *n* = 49) and right ventricular (RV) area (− 3.5 ± 5.2 cm2, 95% CI -5.1, − 1.9; *p* < 0.001; *n* = 44), RV thickness (− 0.76 ± 2.2 mm, 95% CI -1.55, 0.03; *n* = 32), and a significant increase in TAPSE (2.95 ± 4.78 mm, 95% CI 1.52, 4.39; *n* = 45) and RV fractional area change (8.12 ± 8.87 mm, 95% CI 4.61, 11.62; *n* = 27).

Both LOCF and BOCF showed similar results but lower effect sizes.

**Conclusion:**

Patients under long-term treatment with riociguat show significantly reduced right heart size and improved RV function in PAH and CTEPH. Further controlled prospective studies are needed to confirm these results.

## Background

Pulmonary arterial hypertension (PAH) and chronic thromboembolic pulmonary hypertension (CTEPH) are conditions both characterized by a progressive vascular remodeling, increase in pulmonary arterial pressure, pulmonary vascular resistance (PVR) and right ventricular (RV) overload that can ultimately lead to right heart failure and death [1]. At diagnosis most patients show an enlarged right heart with increased RV and right atrial (RA) area (>16cm^2^) and impaired cardiac output (CO). Riociguat is the first drug that has been approved for the treatment of both PAH and CTEPH [2, 3]. In patients with PAH, the 12-week PATENT-1 study showed a significant improvement of the primary endpoint 6-min walking distance (6MWD) and of secondary end-points as PVR, N-terminal prohormone brain natriuretic peptide (NT-proBNP) levels, World Health Organisation functional class (WHO-FC), time to clinical worsening, and Borg dyspnea score [2]. An exploratory analysis of the first 12 weeks of the long-term extension study (PATENT-2) showed further significant improvement of 6-MWD in the 215 PAH-patients receiving up to 2.5 mg of riociguat three times daily [4].

In CTEPH patients the 12-week CHEST-1 study revealed a statistically significant improvement of 6MWD (primary end-point) and in PVR, NT-proBNP serum values, and in WHO-FC (secondary end points) [3]. Further studies investigating the effect of riociguat in an uncontrolled design were the early access study (EAS) in patients with CTEPH [5] and PATENTplus in PAH [1]. Due to potentially unfavourable safety signals with sildenafil plus riociguat in the PATENTplus study and no evidence of a positive benefit/risk ratio, concomitant use of riociguat with phosphodiesterase-5 inhibitors is contraindicated [6].

Sparse data are currently available on the effects of riociguat administration on right heart size and function. RV size and function have not been addressed as end-points in the respective riociguat studies. The study protocols of the phase III riociguat studies did not include echocardiographic assessments to investigate right heart size as RV or RA areas. However, right heart size and function are of utmost prognostic importance in PAH/CTEPH. RV performance measured by echocardiography [7, 8] and enlarged RA area [7, 9] have been shown to be independent prognostic factors in PAH. Recently it has been shown that riociguat treatment (1.0–2.5 mg tid) was associated with a significant reduction of RV and RA area after 3, 6 and 12 months compared to baseline [10]. RA area significantly decreased after 12 months and RV systolic function assessed with tricuspid annular plane systolic excursion (TAPSE) improved after 6 and 12 months of riociguat therapy [10].

The aim of the current study was to assess the effects of long-term riociguat treatment on right heart size and function measured by echocardiography in patients with PAH or CTEPH who have been enrolled in the prospective, randomized, double-blind, multicentre, parallel-group, placebo-controlled riociguat trails PATENT-1, PATENTplus, CHEST-1, phase II trial and Early Access Study and the corresponding long-term extension trials.

## Methods

### Study design

This clinical investigation was performed as a retrospective analysis using the data of prospective, randomized, double-blind, multicentre, parallel-group, placebo-controlled clinical studies (riociguat trails PATENT-1 study, PATENTplus study, CHEST-1 study, phase II trial and Early Access Study) and the corresponding long-term extension trials with add-on of echocardiographic data obtained during routine assessments which have been performed during these trials. All riociguat studies were approved by the ethics committees of the respective centers. The ethics committee of the medical faculty, University of Heidelberg had no objection against the retrospective analysis of echocardiographic data within this study (S668–2015). Due to the small number of patients with available echocardiographic data receiving placebo, the data was evaluated as single armed, observational study.

### Study population

German centres were contacted to include patients who were enrolled within the riociguat phase II and III trials and received routine echocardiographic assessments throughout the study period. In five centres Dresden, Hannover, Heidelberg, Giessen and Regensburg echocardiographic assessments were routinely performed at baseline and after 3, 6 and 12 months beside the parameters which have been obtained in the context of the riociguat trials. All patients with PAH (PATENT-1 and 2, PATENTplus) or CTEPH (CHEST-1 and 2, Early Access Study) randomized into one of the trials (i.e. for whom study medication had been assigned and a package had been opened) and who had at least once been administered study medication were eligible for the study, if echocardiography had been routinely performed at baseline and at least once during the course of the trial.

### Analysis of echocardiography

For analysis of the echocardiographic parameters including RA and RV areas, TAPSE, RV thickness, tricuspid regurgitation velocity, RV fractional area change, right and left ventricular systolic function, left ventricular eccentricity index, pulmonary artery diameter and presence of pericardial effusion we obtained the DICOM-films of the on-line echocardiographic assessments performed in each centre and analysed them off-line in Heidelberg. In case of low image quality and lacking multiple measurements, values were not included in the data base. For the off-line readings specialised software was used (TOMTEC) by an experienced physician. The investigator (AM) who performed the off-line readings of the echocardiography-DICOM-data was blinded to the patient name, treatment group, location and time point of the assessment, demographic and clinical data. All echocardiographic measurements were performed according to American Society of Echocardiography/European Association of Cardiovascular imaging guidelines [11]. Accordingly, RA area and RV area were measured at the end of ventricular diastole (largest volume), tracing following the endocardium from the lateral aspect of the tricuspid annulus to the septal aspect, excluding the area of the annulus and the leaflets (with regard to RA area) and excluding the area of the annulus and trabecular structures (with regard to RV area). RV systolic area was calculated with the same methodology but at the end of the systole. RV fractional area change was defined as: [(RV end-diastolic area – RV end-systolic area)/ RV diastolic area]*100. TAPSE was calculated using M-mode from the lateral tricuspid annulus. Results of off-line readings were forwarded to an external contract research organisation in order to merge with the clinical data of the riociguat trials. A clinically significant improvement of right heart size was defined as a ≥ 15% reduction RA or RV area [12].

### Data collection

Data from the riociguat studies were used for this study to complete clinical characterization of the patient cohort. The respective parameters from the riociguat trials database were merged with the echocardiography data after double pseudonymization for data protection by contract research organisation. Parameters comprised of baseline demographics, vital signs, WHO functional class, hemodynamics, 6-min walking distance, NTpro-BNP and PH targeted treatment.

Patients who received riociguat in the core study were included with their baseline examination of the core study and the subsequent follow-up examinations. Patients who received placebo in the core study and riociguat in the extension study were included with their final examination of the double-blind study (= baseline examination of the extension study) and the subsequent follow-up examinations.

Patients with invalid echocardiographic recordings, insufficient quality of recordings or missing baseline echocardiography were excluded from the analysis.

### Statistics

Descriptive statistics are displayed by mean ± standard deviation, median and interquartile ranges. Frequency tables are provided for qualitative data. The primary endpoint was defined as the change of RA area from baseline to 12 months of treatment. The second part of the primary endpoint with comparison of changes during treatment between riociguat and placebo group was not possible due to the small sample size of the placebo group (*n* = 5). Previous power analysis for the primary endpoint determined a statistical power of at least 87.7% at an alpha level of 0.025 with a sample size of 75 patients if the true treatment effect was a reduction of RA area of at least 3 cm^2^ with a standard deviation of the difference of 7.5 cm^2^ after 12 months of treatment.

For the comparison of baseline and follow up values the t-test for paired measurements or the Wilcoxon signed rank test was used, as appropriate. Frequency distributions between baseline and follow-up were compared with the test of marginal homogeneity if at least one of the variables had more than two different values. For 2 × 2 tables the McNemar test was performed. Comparisons between groups were analysed with the t-test for independent samples or the Mann Whitney U test. For the comparison of frequency distributions between groups Fishers exact test was used.

For missing values of echocardiographic parameters after 12 months, baseline observation carried forward (BOCF) and last observation carried forward (LOCF) methods were performed as sensitivity analysis.

All tests are two-tailed and *p*-values < 0.05 were considered as statistically significant. All analyses were performed with SAS 9.3 for Windows (SAS Institute, Cary NC) and SPSS V 24.

## Results

**Fig. 1:**
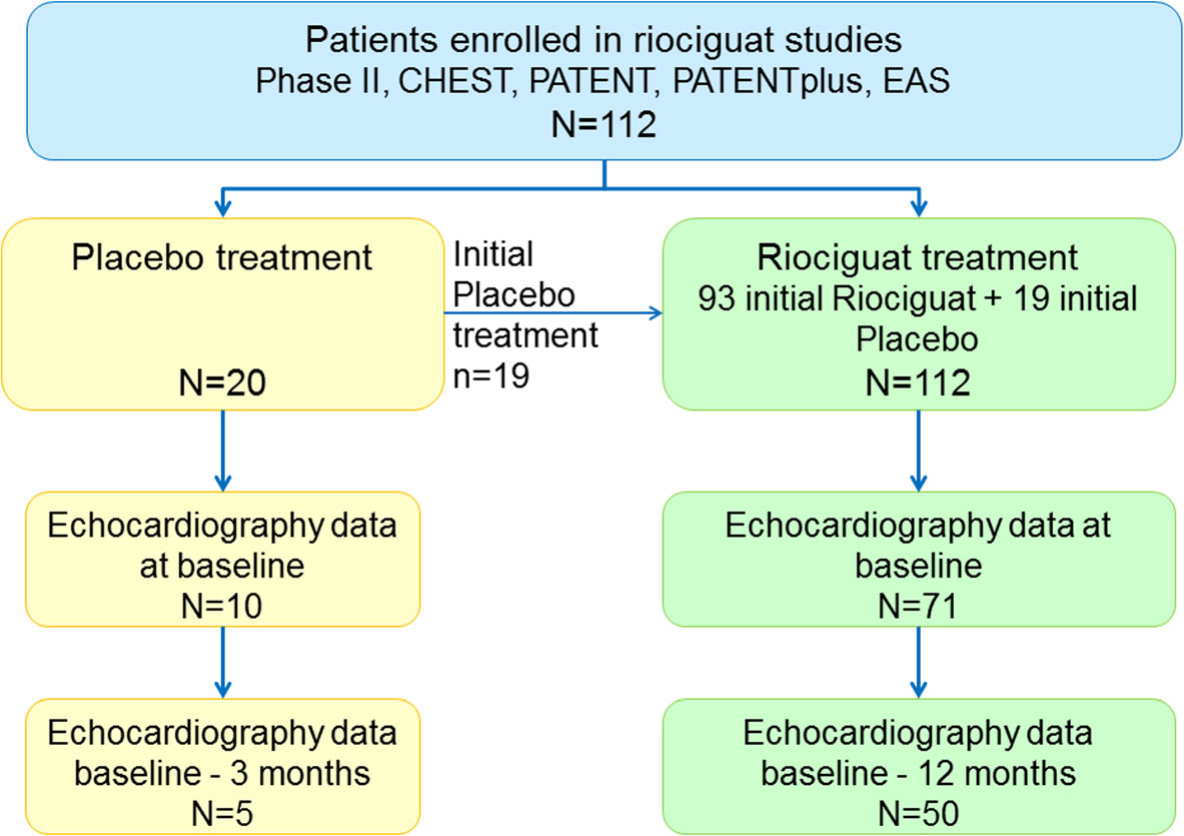
RIVER study Flow-Chart. All patients with PAH (PATENT-1 and 2, PATENTplus) or CTEPH (CHEST-1 and 2, Early Access Study) randomized into one of the trials (i.e. for whom study medication had been assigned and a package had been opened) and who had at least once been administered study medication were eligible for the study, if echocardiography had been routinely performed at baseline and at least once during the course of the trial

Within the riociguat studies the participating centres included 112 patients (55% female, 43% PAH, 60 ± 13 years; 20 patients starting with placebo, 92 with riociguat), who had at least one echocardiographic assessment during the treatment period. In 71 patients (54% female, 83% treatment-naïve, 60 ± 13 years, mean pulmonary arterial pressure 46 ± 10 mmHg; Table 1, Fig. 1) echocardiography was available at the start date of the riociguat treatment. Out of them, 59 started the study with riociguat (83.1% of 71), 12 with placebo (16.9% of 71); the latter were switched to open-label riociguat after 12 and 16 weeks, respectively. In 20 patients who received initial placebo treatment, data from baseline echocardiography was available. Out of them, five patients presented with echocardiographic data after 3 months of placebo treatment. Because of the small and possibly biased placebo group, we did not perform a statistical test for the comparison of both groups.

**Table 1:** Baseline characteristics of the study cohort

		Mean ± standard deviation or n and (%)	95% CI (mean)		median and interquartile ranges
Variable	N		lower limit	upper limit	
Demographics					
Age [years]	71	59.1 ± 13.42	56.0	62.3	60 [50; 71]
Height [cm]	71	167.6 ± 9.89	165.2	169.9	168 [160; 174]
Weight [kg]	71	76.91 ± 17.53	72.77	81.06	73.0 [65; 87]
BMI [kg/m^2^]	71	27.26 ± 5.04	26.06	28.45	26.42 [24; 29.05]
gender [female n (%)]		38 (53.5%)			
Pulmonary hypertension etiology					
Idiopathic PAH		14 (19.7%)			
CTD-APAH		10 (14.1%)			
Other PAH		8 (11.3%)			
Chronic thromboembolic pulmonary hypertension		39 (54.9%)			
WHO functional class					
II		17 (23.9%)			
III		54 (76.1%)			
Pulmonary hypertension targeted treatment					
treatment naÏve		52 (73%)			
Endothelin-Receptor-Antagonist		14 (20%)			
Phosphodiesterase-5-inhibitor		5 (7%)			
Oxygen		7 (10%)			
Vital signs					
Systolic Blood Pressure [mmHg]	71	114.3 ± 12.90	111.2	117.3	110 [105; 123]
Diastolic Blood Pressure [mmHg]	71	72.0 ± 9.93	69.6	74.3	70 [65; 80]
Heart Rate [bpm]	71	80.6 ± 9.99	78.2	83.0	80 [72; 88]
Clinical parameters					
NTproBNP [pmol/L]	41	140.97 ± 230.87	68.10	213.84	43.3 [18.1; 175.5]
6-min walking distance [meters]	68	359.0 ± 91.48	336.9	381.1	359 [295; 419]
Hemodynamics measured by right heart catheter					
mean PAP [mmHg]	45	46.13 ± 9.833	43.17	49.08	46.0 [39; 54]
Pulmonary Vascular Resistance [dyn*sec*cm^−5^]	45	700.75 ± 282.82	615.79	785.72	593.6 [510.6; 829]
Cardiac Index [L/min/m^2^]	45	2.53 ± 0.46	2.39	2.66	2.58 [2.31; 2.78]
Cardiac Output [L/min]	45	4.69 ± 1.06	4.37	5.01	4.6 [4.3; 5.2]
Pulmonary Arterial Wedge Pressure [mmHg]	45	7.4 ± 2.85	6.5	8.2	7 [5; 8]

*CI* confidence interval, *BMI* Body mass index, *PAH* pulmonary arterial hypertension, *CTD* connective tissue disease

*NTproBNP* fragment of N-terminal pro brain natriuretic peptide, *PAP* pulmonary arterial pressure

No patient died throughout the reported study period. Two patients who were not included in the analysis (due to missing echocardiographic assessment) received a lung transplantation.

### Effect of riociguat on right heart size and function

**Fig. 2:**
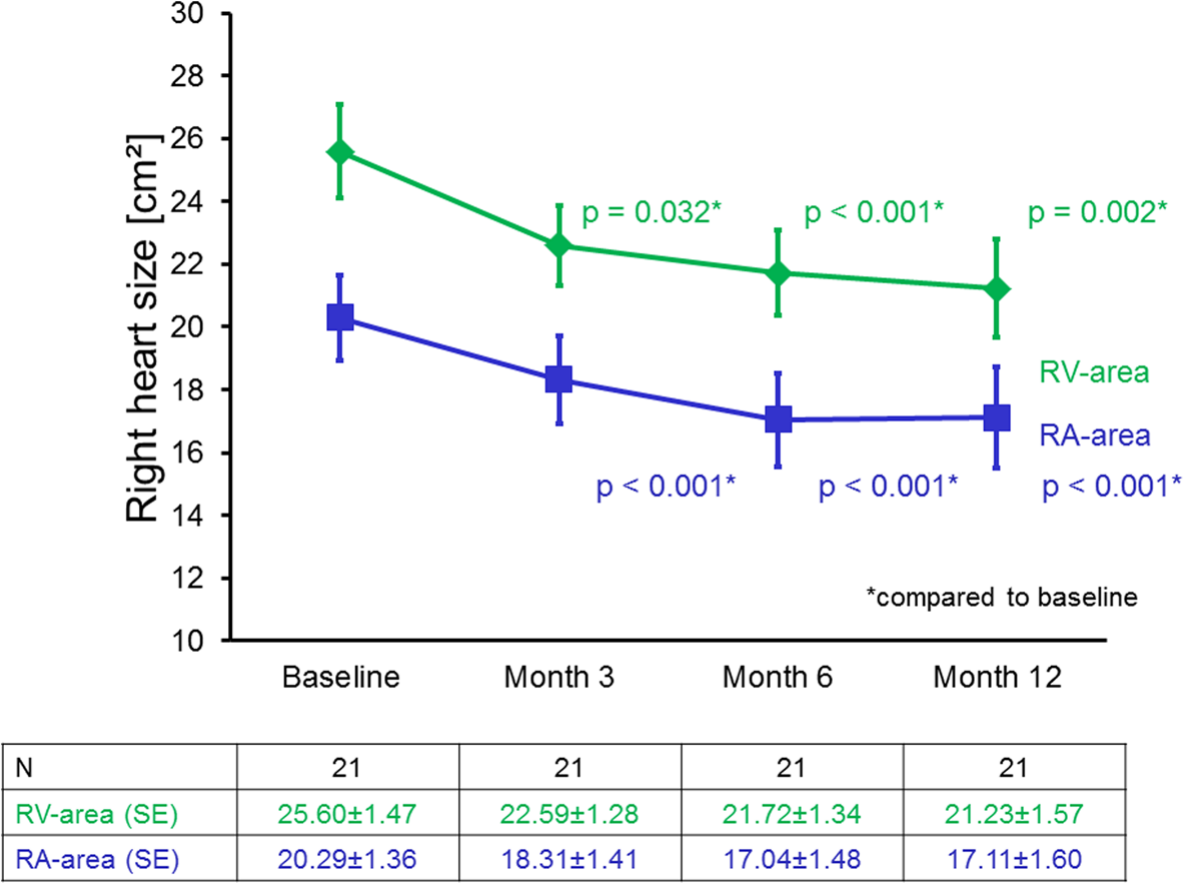
Right atrial and right ventricular areas in patients who completed all four echocardiographic assessments. Patients who completed echocardiographic assessments at baseline, 3, 6 and 12 months showed a significant reduction of right atrial and right ventricular areas for all time points. Values are given as mean ± standard error of the mean

Imaging quality was very good in > 90% of cases. In 71 patients with baseline-echocardiography, changes of echocardiographic parameters after three (*n* = 30), six (*n* = 34) and 12 months (*n* = 50) are shown in Table 2. Patients receiving riociguat therapy showed a significant reduction in RA area after 6 (− 2.4 ± 3.7 cm^2^, 95% confidence interval (CI) -3.73, − 1.13; *p* < 0.001, *n* = 34) and 12 months (− 2.6 ± 4.4 cm^2^, 95% CI -3.84, − 1.33; *p* < 0.001, *n* = 49). Likewise, patients showed a significant decrease in RV area (3 months: − 3.0 ± 3.0 cm^2^, 95% CI -4.1, − 1.9; 6 months:− 3.0 ± 3.15 cm^2^, 95% CI -4.23, − 1.96; 12 months: − 3.5 ± 5.2 cm^2^, 95% CI -5.1, − 1.9; *p* < 0.001 for all time points; *n* = 29, 32, 44, respectively). Patients who completed all four echocardiographic assessments (at baseline, after 3, 6 and 12 months (*n* = 21)) presented with a significant reduction of RA and RV areas for all time points (Table 2, Fig. 2).

**Table 2:** Changes of echocardiographic parameters after 3, 6 and 12 months of riociguat treatment

	baseline - 3 months						baseline - 6 months						12 months					
		Mean ± standard deviation	95% CI (mean)		median and interquartile ranges			Mean ± standard deviation	95% CI (mean)		median and interquartile ranges			Mean ± standard deviation	95% CI (mean)		median and interquartile ranges	
	N		lower limit	upper limit		*p*-value	N		lower limit	upper limit		*p*-value	N		lower limit	upper limit		*p*-value
RA area [cm^2^]	30	- 1.21 ± 3.94	- 2.67	0.26	1.0 [−4.54; 1.69]	0.104	34	- 2.43 ± 3.74	- 3.73	- 1.13	1.99 [−5.0; 0.29]	< 0.001	49	- 2.58 ± 4.37	- 3.84	- 1.33	2.38 [−6.0; 0.3]	< 0.001
RV area [cm^2^]	29	-3.0 ± 2.95	−4.12	−1.87	2.0 [−5.0; − 1.0]	< 0.001	32	−3.01 ± 3.15	−4.23	−1.96	−2.36 [−5.5; − 1.0]	< 0.001	44	- 3.51 ± 5.23	−5.1	− 1.92	− 3.25 [− 5.6; 0.0]	< 0.001
TAPSE [mm]	29	0.86 ± 2.97	- 0.27	1.99	0 [−1.0; 2.0]	0.257	32	2.38 ± 4.25	0.85	3.92	2.0 [0; 3.7]	0.003	45	2.95 ± 4.78	1.52	4.39	3.7 [0; 5.5]	< 0.001
FAC [mm]	15	1.67 ± 3.18	−0.09	3.43	0.99 [0; 2.41]	0.068	16	6.13 ± 4.32	3.83	8.43	5.11 [3.69; 8.33]	< 0.001	27	8.12 ± 8.87	4.61	11.62	8.72 [−3.2; 15.0]	< 0.001
RV thickness [mm]	28	−0.68 ± 1.99	−1.45	0.1	0.0 [0.0; 1.75]	0.109	27	−1.13 ± 2.03	−1.94	−0.33	1.0 [0.0; 2.0]	0.008	32	−0.76 ± 2.2	−1.55	0.03	0.9 [0.0; 2.0]	0.023
TRV [cm/sec]	29	- 0.28 ± 0.5	−0.47	−0.09	−0.24 [− 0.4;-0.02]	0.005	32	− 0.48 ± 0.63	−0.71	− 0.25	−0.57 [− 0.71; − 0.07]	< 0.001	47	−0.36 ± 0.83	−0.6	−0.12	− 0.52 [− 0.77; − 0.03]	0.005
IVC diameter [mm]	22	−0.81 ± 3.48	−2.35	0.73	0.5 [−2.0; 0.0]	0.288	25	−0.23 ± 3.83	−1.81	1.35	0.0 [− 2.0; 3.0]	0.764	28	−0.50 ± 4.07	−2.08	1.08	1.0 [−3.45; 2.6]	0.518
LV-EI	27	−0.05 ± 0.17	−0.12	0.02	0.0 [−0.1; 0.0]	0.147	30	−0.005 ± 0.25	−0.1	0.09	0.0 [−0.2; 0.1]	0.906	40	−0.13 ± 0.22	−0.20	−0.06	− 0.1 [− 0.25; 00]	< 0.001
PA diameter [mm]	21	− 0.05 ± 2.09	−1.0	0.9	0.0 [−1.0; 0.0]	0.886	21	−0.99 ± 4.35	−2.97	0.99	0.0 [−2.0; 0.7]	0.309	26	−1.64 ± 3.17	−2.92	0.36	−1.0 [−3.5; 0.0]	0.014

*TAPSE* tricuspid annular plane systolic excursion, *FAC* Fractional area change, *RA* right atrial, *RV* right ventricular, *TRV* tricuspid regurgitation velocity, *IVC* inferior vena cava, *LV-EI* left ventricular eccentricity index

*PA* pulmonary artery

After three months seven of 30 patients had a marked reduction in RA area of ≥15% compared to baseline (23.3% of 30); after six months 14 patients reached this reduction (41.2% of 34) and after 12 months 23 patients (46.9% of 49) (Table 2).

At baseline 20 of 70 patients showed a RA area lower than 18cm^2^ (28.6% of 70, one patient with baseline echocardiography had a missing RA area value). After 3 months 15 had a RA area < 18 cm^2^ (50.0% of 30), after 6 months 18 (52.9% of 34) and after 12 months 25 (51.0% of 49), respectively (Table 2).

After 12 months of riociguat treatment patients showed a significant decrease in RV thickness (− 0.76 ± 2.2 mm, 95% CI -1.55, 0.03), tricuspid regurgitation velocity (− 0.36 ± 0.83 cm/sec, 95% CI -0.6, − 0.12), pulmonary artery diameter (− 1.64 ± 3.17 mm, 95% CI -2.92, 0.36) and a significant increase in TAPSE (2.95 ± 4.78 mm, 95% CI 1.52, 4.39) and RV fractional area change (8.12 ± 8.87 mm, 95% CI 4.61, 11.62) (Table 2).

Patients also presented with an improvement of qualitatively assessed RV systolic function after 12 months of treatment (normal systolic function at baseline *n* = 6 vs. 16 at 12 months, mild impairment 15 vs. 16, moderate impairment 15 vs. 7, severe impairment 9 vs. 6; χ^2^-test *p* = 0.016). Left ventricular systolic function and presence of pericardial effusion remained stable throughout the study period. Both LOCF and BOCF showed similar significance and lower effect sizes (data not shown).

After 12 months of riociguat treatment patients showed a significant increase in mean 6MWD (59.6 ± 81 m, *p* < 0.001).

### Description of patients who did not receive echocardiography after 12 months

Echocardiography after 12 months riociguat treatment was performed for 50 out of the 71 riociguat patients with echocardiography at riociguat start (70.4% of 71), 21 patients had no echocardiography after 12 months (29.6% of 71).

Patients who did not complete their 12-month echocardiographic assessment did not significantly differ regarding gender, age, height, weight, severity of the disease, 6MWD and hemodynamics from patients who received an echocardiographic examination after 12 months (Table 3, all *p* > 0.05). Baseline values for 6MWD and cardiac index were lower, pulmonary vascular resistance and NT-proBNP higher in patients who did not complete their 12 month examination, though differences between the two groups did not reach statistical significance (Table 3). Median values for PVR, cardiac index and cardiac output were similar between groups.

**Table 3:** Comparison of patients with echocardiography and patients with missing examination after 12 months

Variable	12 months dropouts [*n* = 21]					12 months completers [*n* = 50]					
		Mean ± standard deviation or n and (%)	95% CI (mean)		median and interquartile ranges		Mean ± standard deviation or n and %	95% CI (mean)		median and interquartile ranges	
	N		lower limit	upper limit		N		lower limit	upper limit		*p*-value
Demographics											
Age [years]	21	59.5 ± 11.66	54.2	64.8	56 [49; 71]	50	59.0 ± 14.2	54.9	63.0	60 [51; 71]	0.845
Height [cm]	21	170.1 ± 10.95	165.1	175.1	172 [163; 175]	50	166.5 ± 9.3	163.9	169.2	168 [160; 173]	0.267
Weight [kg]	21	78.7 ± 12.5	73.0	84.4	75.0 [71; 88]	50	76.2 ± 19.3	70.7	81.6	71 [65; 85]	0.196
BMI [kg/m^2^]	21	27.2 ± 3.5	25.6	28.8	26.8 [25.7; 28.4]	50	27.3 ± 5.6	25.7	28.9	26.0 [23.3; 30.0]	0.575
gender [female n (%)]		11 (52.4%)					27 (54.0%)				
Pulmonary hypertension etiology											
Idiopathic PAH		3 (14.3%)					11 (22.0%)				
Other PAH		6 (28.6%)					12 (24.0%)				
Chronic thromboembolic pulmonary hypertension		12 (57.1%)					27 (54.0%)				
WHO functional class											
II		6 (28.6%)					11 (22.0%)				
III		15 (71.4%)					39 (78.0%)				
Pulmonary hypertension targeted treatment											
treatment naÏve		2 (33.3%)					35 (70%)				
Endothelin-Receptor-Antagonist		2 (33.3%)					12 (24%)				
Phosphodiesterase-5-inhibitor		2 (33.3%)					3 (6%)				
Oxygen		2 (33.3%)					5 (10%)				
Vital signs											
Systolic Blood Pressure [mmHg]	21	116 ± 12	110	121	110 [110; 125]	50	114 ± 14	110	121	110 [110; 125]	0.378
Diastolic Blood Pressure [mmHg]	21	75 ± 10	71	80	76 [70; 80]	50	71 ± 10	68	73	70 [64; 80]	0.040
Heart Rate [bpm]	21	79 ± 9	75	83	80 [72; 84]	50	81 ± 11	78	84	80 [73; 88]	0.645
Clinical parameters											
NTproBNP [pmol/L]	11	183.7 ± 243.4	20.2	347.2	76 [29.6; 217.4]	30	125.3 ± 228.4	40.0	210.6	42.2 [17.4; 144.8]	0.471
6-min walking distance [meters]	18	338.5 ± 99.9	288.8	388.2	362 [270; 406]	50	366.4 ± 88.1	341.3	391.4	370 [301; 420]	0.440
Hemodynamics measured by right heart catheter											
mean PAP [mmHg]	13	48.6 ± 54.1	43.0	54.1	47 [45; 57]	32	45.1 ± 10.1	41.5	48.8	43.5 [37.8; 53.3]	0.215
Pulmonary Vascular Resistance [dyn*sec*cm^− 5^]	13	781.3 ± 259.3	624.6	938.0	747.8 [615.4; 904.3]	32	668.0 ± 289.3	563.8	772.3	563.1 [495.2; 789.7]	0.112
Cardiac Index [L/min/m^2^]	13	2.4 ± 0.5	2.1	2.7	2.5 [2.2; 2.7]	32	2.6 ± 0.5	2.4	2.7	2.6 [2.4; 2.8]	0.276
Cardiac Output [L/min]	13	4.6 ± 0.9	4.0	5.1	4.6 [4.3; 5.2]	32	4.8 ± 1.1	4.3	5.2	4.6 [4.3; 8.2]	0.861
Pulmonary Arterial Wedge Pressure [mmHg]	13	6.0 ± 2.7	4.4	7.6	6 [5; 8]	32	7.9 ± 2.8	6.9	8.9	7 [6; 10]	0.052

*CI* confidence interval, *BMI* Body mass index, *NTproBNP* N-terminal fragment of pro brain natriuretic peptide, *PAP* pulmonary arterial pressure

Patients with 12-month assessment showed a statistically significant but clinically not relevant lower diastolic blood pressure and higher PAWP (Table 3).

### Placebo group

In 20 patients who received placebo treatment, data from baseline echocardiography was available. Out of them, five patients presented with echocardiographic data after 3 months. Patients who received placebo treatment had a mild increase in NT-proBNP (22 ± 104 pmol/l) and 6MWD (18 ± 55 m).

Due to the small sample size of five patients who received an echocardiographic assessment after 3 months, the effect of riociguat was not tested for statistical significance against the control group. Data of patients receiving placebo treatment are presented descriptively (Table 4). RA area slightly decreased within 3 months (0.4 ± 2.9), whereas RV area increased by 1.9 ± 6.4 cm^2^. During the three month period, an increase was seen in TAPSE (0.5 ± 3.2 mm) and RV thickness (1 ± 0.8 mm). Median values of differences between baseline and 3 months did not reveal any marked changes. Presence of pericardial effusion and RV function also remained constant within the 3 months study period.

**Table 4:** Changes of echocardiographic parameters after 3 months of placebo treatment

		Mean ± standard deviation	95% CI (mean)		median and interquartile ranges
Parameter	N		lower limit	upper limit	
Echocardiography					
right atrial area [cm^2^]	5	−0.4 ± 2.9	−4.1	3.2	1 [−3.2; 2]
right ventricular area [cm^2^]	5	2.0 ± 6.4	−6.0	9.8	0 [−5.3; 10]
TAPSE [mm]	5	0.5 ± 3.3	−3.6	4.5	0 [−2.6; 6]
RV thickness [mm]	4	1.0 ± 0.8	−0.3	2.3	1.0 [0.5; 1.5]
TRV [cm/sec]	4	0.1 ± 0.2	−0.3	0.4	0.1 [−0.1; 0.3]
IVC diameter [mm]	5	−0.4 ± 2.7	−3.8	2.9	0 [0; 0.8]
LV-EI	5	−0.1 ± 0.1	−0.2	0.03	−0.1 [− 0.2; 0]
PA diameter [mm]	4	−0.25 ± 1.3	−2.3	1.8	0 [−1.0; 0.5]

TAPSE = tricuspid annular plane systolic excursion; FAC = Fractional area change; RV = right ventricular

*TRV* tricuspid regurgitation velocity, *IVC* inferior vena cava, *LV-EI* left ventricular eccentricity index

*PA* pulmonary artery

## Discussion

To the best of our knowledge this is the first multicentre study showing a reduction of right heart size and improvement of right heart function during treatment with riociguat in patients with PAH or CTEPH. After 6 and 12 months of riociguat treatment, RV and RA area assessed by echocardiography decreased. At the same time, RV systolic function and RV hypertrophy improved. These results were obtained by blinded off-line readings of echocardiographic assessments which have been performed within riociguat trials.

### Relevance of right heart size and function in pulmonary hypertension

These data confirm the results of a recent unblinded single-center study [10] which showed a significant improvement of right ventricular area already after 3 months, an improvement of RV systolic function after 6 months and right atrial area after 12 months treatment. In the current study, RA area was already significantly improved after 6 months of riociguat treatment.

As the current guidelines on treatment goals in pulmonary hypertension state, RA area seems to be one of the most robust echocardiographic determinants of outcome [13] and strongly correlates with RV function [9]. Enlarged RA assessed by transthoracic echocardiography was significantly associated with lower transplant-free survival [7, 14] whereas RA area < 18 cm^2^ was associated with a good prognosis [9, 14]. In this study a reduction of RA area ≥ 15% was achieved by almost 50% of the patients. It has been shown previously that a therapy response to PH targeted medication of ≥15% in RA and RV area may predict long-term outcome in patients with PH [12]. Using the proposed cut-off for a normal RA area of 15cm^2^ in female and 16 cm^2^ in males [15], 31% of patients who presented with baseline values above these cut-offs showed values within the normal ranges after one year of riociguat treatment.

A reduction of right heart size might be an indicator for an improvement of systolic function and reduction in volume overload. In this study the reduction of right heart size was accompanied by a significant improvement of RV systolic function assessed by RV fractional area change, TAPSE and qualitative assessment of RV systolic function.

Preclinical studies showed that Riociguat has a positive effect on pulmonary pressures [16], and is able to reverse RV remodeling [17]. In our study, riociguat administration was associated with an improvement of both RV systolic function, as well as of pulmonary pressures (TRV). The investigation of the underlying predominant mechanisms of riociguat treatment was however out of the scope of our study.

### Strengths and limitations

A strength of this study is the central off-line reading of echocardiographic data by a blinded experienced physician which standardized the interpretation of data and increases its quality. In addition, patients included in this analysis were enrolled in prospective randomized controlled riociguat trials, leading to a thorough and homogenous patient selection (due to similar in- and exclusion criteria of the trials) and high quality of clinical data. As the echocardiographic data derived from routine echocardiographic assessments and were assessed according to the local standards of the participating centres, the study results represent data from clinical practice.

On the other hand, the rate of patients who did not receive echocardiographic assessments after 12 months may have biased the results. However, sensitivity analyses of echocardiographic parameters after 12 months with BOCF and LOCF led to similar significant results with lower effect sizes (data not shown).

Further study limitations are the retrospective nature of the study, limited number of study centres and a small number of patients, in particular those receiving placebo treatment. A higher number of patients receiving riociguat would also have been desirable in order to compare the effects on patients with PAH vs. CTEPH.

## Conclusion

This study suggests that treatment with riociguat may improve right heart function in patients with PAH and CTEPH within 12 months of treatment. Prospective, controlled studies are needed to confirm the effects of riociguat on right heart size and function in these patients.

## Abbreviations

6MWD: 6-min walking distance
95% CI: 95% confidence interval
BOCF: Baseline observation carried forward
CI: Cardiac index
CO: Cardiac output
CTEPH: Chronic thromboembolic pulmonary hypertension
LOCF: Last observation carried forward
mPAP: Mean pulmonary arterial pressure
NT-proBNP: N-terminal pro brain natriuretic peptide
NT-proBNP: N-terminal pro brain natriuretic peptide
PAH: Pulmonary arterial hypertension
PAWP: Pulmonary arterial wedge pressure
PH: Pulmonary hypertension
PVR: Pulmonary vascular resistance
RA: Right atrial
RV: Right ventricular
TAPSE: Tricuspid annular plane systolic excursion
WHO-FC: WHO-functional class

## References

[1] Galiè N, Humbert M, Vachery JL (2015). 2015 ESC/ERS guidelines for the diagnosis and treatment of pulmonary hypertension: The Joint Task Force for the Diagnosis and Treatment of Pulmonary Hypertension of the European Society of Cardiology (ESC) and the European Respiratory Society (ERS): Endorsed by: Association for European Paediatric and Congenital Cardiology (AEPC), International Society for Heart and Lung Transplantation (ISHLT). Eur Respir J.

[2] Ghofrani HA, Galie N, Grimminger F (2013). Riociguat for the treatment of pulmonary arterial hypertension. N Engl J Med.

[3] Ghofrani HA, D'Armini AM, Grimminger F (2013). Riociguat for the treatment of chronic thromboembolic pulmonary hypertension. N Engl J Med.

[4] LJ R, N G, F G, E G, M H, ZC J, A K, D L, A F, F M, N D, Ghofrani HA (2015). Riociguat for the treatment of pulmonary arterial hypertension: a long-term extension study (PATENT-2). Eur Respir J.

[5] McLaughlin VV, Jansa P, Nielsen-Kudsk JE, Halank M, Simonneau G, Grünig E, Ulrich S, Rosenkranz S, Gómez Sánchez MA, Pulido T, Pepke-Zaba J, Barberá JA, Hoeper MM, Vachiéry JL, Lang I, Carvalho F, Meier C, Mueller K, Nikkho S, D'Armini AM (2017). Riociguat in patients with chronic thromboembolic pulmonary hypertension: results from an early access study. BMC Pulm Med.

[6] Galiè N, Müller K, Scalise AV, Grünig E (2015). PATENT PLUS: a blinded, randomised and extension study of riociguat plus sildenafil in pulmonary arterial hypertension. Eur Respir J.

[7] Raymond RJ, Hinderliter AL, Willis PW, Ralph D, Caldwell EJ, Williams W, Ettinger NA, Hill NS, Summer WR, de Boisblanc B, Schwartz T, Koch G, Clayton LM, Jöbsis MM, Crow JW, Long W (2002). Echocardiographic predictors of adverse outcomes in primary pulmonary hypertension. J Am Coll Cardiol.

[8] Bossone E, D'Andrea A, D'Alto M (2013). Echocardiography in pulmonary arterial hypertension: from diagnosis to prognosis. J Am Soc Echocardiogr.

[9] Austin C, Alassas K, Burger C, Safford R, Pagan R, Duello K (2015). Echocardiographic assessment of estimated right atrial pressure and size predicts mortality in pulmonary arterial hypertension. Chest.

[10] Marra AM, Egenlauf E, Ehlken N (2015). Change of right heart size and function by long-term therapy with riociguat in patients with pulmonary arterial hypertension and chronic thromboembolic pulmonary hypertension. Int J Cardiol.

[11] Rudski LG, Lai WW, Afilalo J, Hua L, Handschumacher MD, Chandrasekaran K, Solomon SD, Louie EK, Schiller NB (2010). Guidelines for the echocardiographic assessment of the right heart in adults: a report from the American Society of Echocardiography endorsed by the European Association of Echocardiography, a registered branch of the European Society of Cardiology, and the Canadian Society of Echocardiography. J Am Soc Echocardiogr.

[12] Sano H, Tanaka H, Motoji Y, Fukuda Y, Mochizuki Y, Hatani Y, Matsuzoe H, Hatazawa K, Shimoura H, Ooka J, Ryo-Koriyama K, Nakayama K, Matsumoto K, Emoto N, Hirata KI (2017). Right ventricular relative wall thickness as a predictor of outcomes and of right ventricular reverse remodeling for patients with pulmonary hypertension. Int J Cardiovasc Imaging.

[13] McLaughlin VV, Gaine SP, Howard LS, Leuchte HH, Mathier MA, Mehta S, Palazzini M, Park MH, Tapson VF, Sitbon O (2013). Treatment goals of pulmonary hypertension. J Am Coll Cardiol.

[14] Bustamante-Labarta M, Perrone S, De La Fuente RL, Stutzbach P, De La Hoz RP, Torino A (2002). Right atrial size and tricuspid regurgitation severity predict mortality or transplantation in primary pulmonary hypertension. J Am Soc Echocardiogr.

[15] Grünig E, Henn P, D'Andrea A (2013). Reference values for and determinants of right atrial area in healthy adults by 2-dimensional echocardiography. Circ Cardiovasc Imaging.

[16] Lang M, Kojonazarov B, Tian X (2012). The soluble guanylate cyclase stimulator riociguat ameliorates pulmonary hypertension induced by hypoxia and SU5416 in rats. PLoS One.

[17] Schermuly RT, Stasch JP, Pullamsetti SS (2008). Expression and function of soluble guanylate cyclase in pulmonary arterial hypertension. Eur Respir J.

